# AAV vectors accumulate in the pineal gland after injections into the brain or spinal cord

**DOI:** 10.1016/j.omtm.2021.09.016

**Published:** 2021-10-05

**Authors:** Oswald Steward, Aminata P. Coulibaly, Mariajose Metcalfe, Jamie M. Dam, Kelly M. Yee

**Affiliations:** 1 Reeve-Irvine Research Center, 837 Health Sciences Drive, University of California Irvine School of Medicine, Irvine, CA 92697, USA; 2 Department of Anatomy & Neurobiology, University of California Irvine School of Medicine, Irvine, CA 92697, USA; 3 Department of Neurobiology & Behavior, University of California Irvine School of Medicine, Irvine, CA 92697, USA; 4 Department of Neurosurgery, University of California Irvine School of Medicine, Irvine, CA 92697, USA

**Keywords:** adeno-associated virus, AAV vector, pineal gland

## Abstract

AAV vectors are being used extensively for gene-modifying therapies for neurological disorders. Here, we report the surprising discovery that injections of different AAVs into the brain, spinal cord, or cerebrospinal fluid (CSF) lead to robust transduction of cells in the pineal gland. We document transduction of cells in the pineal gland following focal injections of AAV2/9-shPTEN-zsGreen into the sensorimotor or hippocampus of rats and injections of AAV2/Cre into the spinal cord of transgenic mice with a stop-flox tdT reporter. Pineal transduction was evident even when AAV2/Cre injections were made into the lumbar spinal cord many millimeters distant from the pineal gland. Immunostaining with antibodies for cell types in the pineal gland revealed that pinealocytes were transduced. Pineal transduction was also observed with intracerebroventricular (i.c.v.) injections of AAV2/9-shPTEN-zsGreen, suggesting that pineal transduction following focal injections of AAV into CNS parenchyma may be caused by diffusion of the vector from the injection sites into the CSF and then accumulation in the pineal gland. Together, these findings suggest the need for vigilance for functional consequences and possible adverse effects of off-target accumulation of therapeutic AAVs in the pineal gland and AAV-driven expression of therapeutic cargos in pinealocytes.

## Graphical abstract

**Figure:**
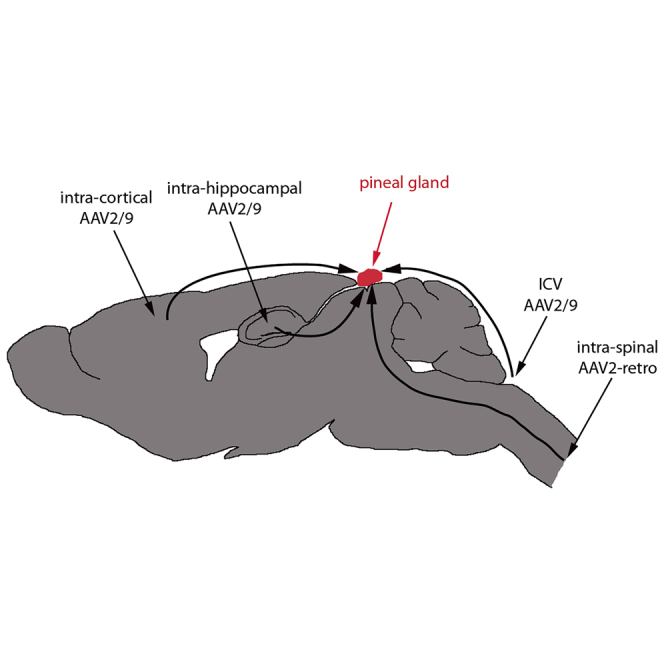

## Teaser

We report the surprising discovery that different AAVs injected into the brain, spinal cord, or cerebrospinal fluid (CSF) accumulate in the pineal gland and transduce pinealocytes. These findings suggest the need for vigilance for functional consequences and possible adverse effects of pineal transduction by therapeutic AAVs injected into the CNS.

## Introduction

AAV-based gene-modifying vectors are rapidly advancing as candidate therapies for a range of neurological disorders.^1^ The recent dramatic clinical success and rapid US Food and Drug Administration (FDA) approval of Zolgensma for spinal muscular atrophy (SMA) is one example, and others are in the translational pipeline, including other trials targeting genetic deficiencies in SMA types 1 and 2 (ClinicalTrials.gov: NCT02122952 and NCT03381729) and Batten disease (ClinicalTrials.gov: NCT02725580). Several of these therapeutic candidates are based on AAV9 because of its capacity for transducing neurons and glia throughout the brain, but therapies involving other AAV serotypes are also in the pipeline.

Translational programs for biologicals require assessments of biodistribution and potential off-target effects; these are safety considerations for any therapeutic agent and particularly for gene-modifying AAV vectors. Biodistribution assays routinely assess a variety of tissues; however, to our knowledge, the pineal gland is not normally included.

In the course of studies using AAVs to transduce neurons to enable regeneration after spinal cord injury, we noticed an unexpected accumulation of AAV2/9 and AAV2 vectors in the pineal gland after focal injections into sites in the brain and spinal cord. We mentioned this incidental observation in a single sentence in a previous study,^2^ and here we report our findings in detail.

The functions of the pineal gland are incompletely understood, so potential consequences of AAV transduction and the resulting expression of gene cargos are not obvious and will depend on the cargos that are expressed. Thus, our findings of unexpected off-target sites of transduction raise questions about potential safety concerns going forward and suggest the need for including the pineal gland in routine biodistribution screens along with further studies of functional consequences and possible adverse effects of AAV-driven expression of therapeutic genes in pinealocytes.

## Results

### Accumulation of AAV in the pineal gland with AAV injections into the sensorimotor cortex

Our initial discovery of accumulation of AAV in the pineal gland was in a study in which AAV2/9-shPTEN-zsGreen was injected into the sensorimotor cortex in rats in order to knock down PTEN in cortical motoneurons to promote regeneration after spinal cord crush injuries at thoracic level 9 (T9). Some rats also received transplants of GFP-labeled neural stem cells (NSCs) into the injury cavity. Hindlimb locomotor function was tested over a 4-month survival interval, and rats were then perfused for histology.

**Figure 1:**
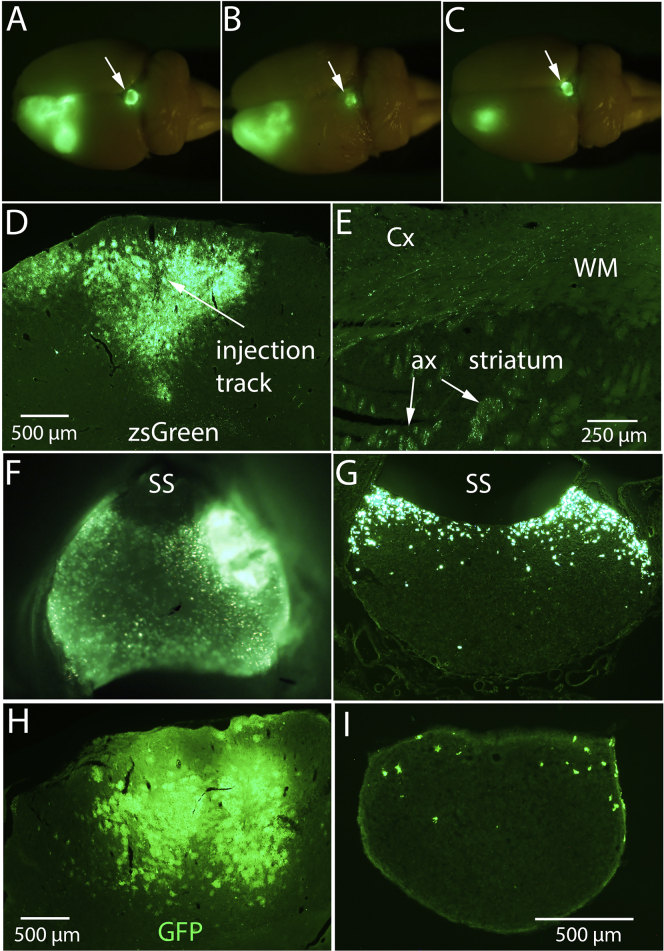
Transduction of cells in the pineal gland in rats with cortical injections of AAV2/9-shPTEN-zsGreen or AAV2/9-shPTEN-GFP (A–C) Fluorescence epi-illumination of intact brain from three rats that received injections of AAV2/9-shPTEN-zsGreen into the sensorimotor cortex. Arrows indicate the pineal gland. (D) zsGreen fluorescence in a coronal section through the injection site. (E) zsGreen-labeled axons in the subcortical white matter extending several millimeters from the injection site, and collections of zsGreen-labeled axons in the dorsal striatum (arrows). (F) High-magnification view of pineal gland still attached to an intact brain reveals thousands of zsGreen-positive cells. (G) zsGreen-positive cells in a section through the most rostral part of the pineal gland shown in (F). (H) GFP immunofluorescence in a coronal section through the injection site from a rat that received injections of AAV2/9-shPTEN-GFP into the sensorimotor cortex. (I) GFP-positive cells in a section through the pineal gland. Calibration bar in (I) applies to (F), (G), and (I). ax, axons; WM, white matter.

As a quick and convenient way to determine the survival of the GFP-positive NSC transplants in the spinal cord and scan for any ectopic stem cell colonies that were present, we used a Night Sea flashlight to illuminate intact brains and spinal cords prior to histological preparation. In addition to revealing the transplants, there was striking zsGreen fluorescence in the area of the injection site in the cortex revealing the area of AAV transduction. We were surprised, however, to discover that the pineal gland was also brightly fluorescent in many rats at an apparent wavelength matching that of zsGreen rather than the GFP expressed by NSCs. Examples of three cases are illustrated in Figures 1A–1C.

The pineal gland is closely associated with the dura mater and often detaches from the brain when removing the dura mater, and in the above study, we had not taken the extra care necessary during removal of the brains from the skull to keep the pineal gland attached. Consequently, the pineal gland remained attached in 10/20 brains. zsGreen fluorescence was present in all 10 of these pineal glands. In five rats, part of the pineal gland remained attached to the brain, and zsGreen was present in the residual tissue of all of these. In the remaining five rats in which the pineal gland was lost during brain removal, no zsGreen fluorescence was evident in the region where the pineal gland had been connected.

To further define the incidence of pineal transduction, we assessed zsGreen expression in pineal glands in another set of rats from a study of consequences of PTEN knockdown on activity-dependent immediate early gene (IEG) expression in neurons.^3^ Ten rats received AAV2/9-shPTEN-zsGreen injections into the sensorimotor cortex as above and 10 received a control vector (AAV2/9-shLuc-zsGreen), and rats were allowed to survive for 2 months post-injection. Fluorescence epi-illumination of intact brains prior to sectioning revealed zsGreen fluorescence in the pineal gland in 7 of the 10 brains that received AAV2/9-shPTEN-zsGreen. Pineal glands were lost during brain removal in three rats. Similarly, zsGreen fluorescence was present in the pineal gland in 8/10 brains that received AAV2/9-shLuc-zsGreen, and pineal glands were lost during brain removal in two rats. Thus, taken together, zsGreen fluorescence was present in the pineal gland in 15/15 brains in which at least part of the pineal gland remained attached.

Examination of coronal sections through the injection site revealed that, as expected, zsGreen fluorescence in the cortex was concentrated at the injection sites (Figure 1D). There were a few cells with the morphology of astrocytes in the cortex lateral to the injection core (more on this below). zsGreen labeling was mostly restricted to the cortical gray matter, although labeled axons extended into the subcortical white matter and into the subjacent striatum (Figure 1E, ax: arrows).

#### Source of zsGreen fluorescence in the pineal gland

To further explore this phenomenon, we followed up with more detailed studies of pineal gland tissue from the above studies and from other sets of rats. One set of 50 rats included 26 that received spinal cord injuries and intra-cortical injections of AAV2/9-shPTEN-zsGreen and 24 that received AAV2/9-shLuc-zsGreen as above. This set of 50 rats survived for 4 months post-injection. Another set of six rats received intra-cortical injections of AAV2/9-shPTEN-GFP (same injection parameters as above) and survived for 3 months post-injection.

We first explored the source of the zsGreen fluorescence in the pineal glands. Even with epifluorescence imaging of pineal glands still attached to intact brains, it is evident that the fluorescence originates from strongly fluorescent cell bodies (Figure 1F). In coronal sections through this pineal gland, large numbers of zsGreen-labeled cells are evident (Figure 1G, see below for further quantitative analysis).

**Figure 2:**
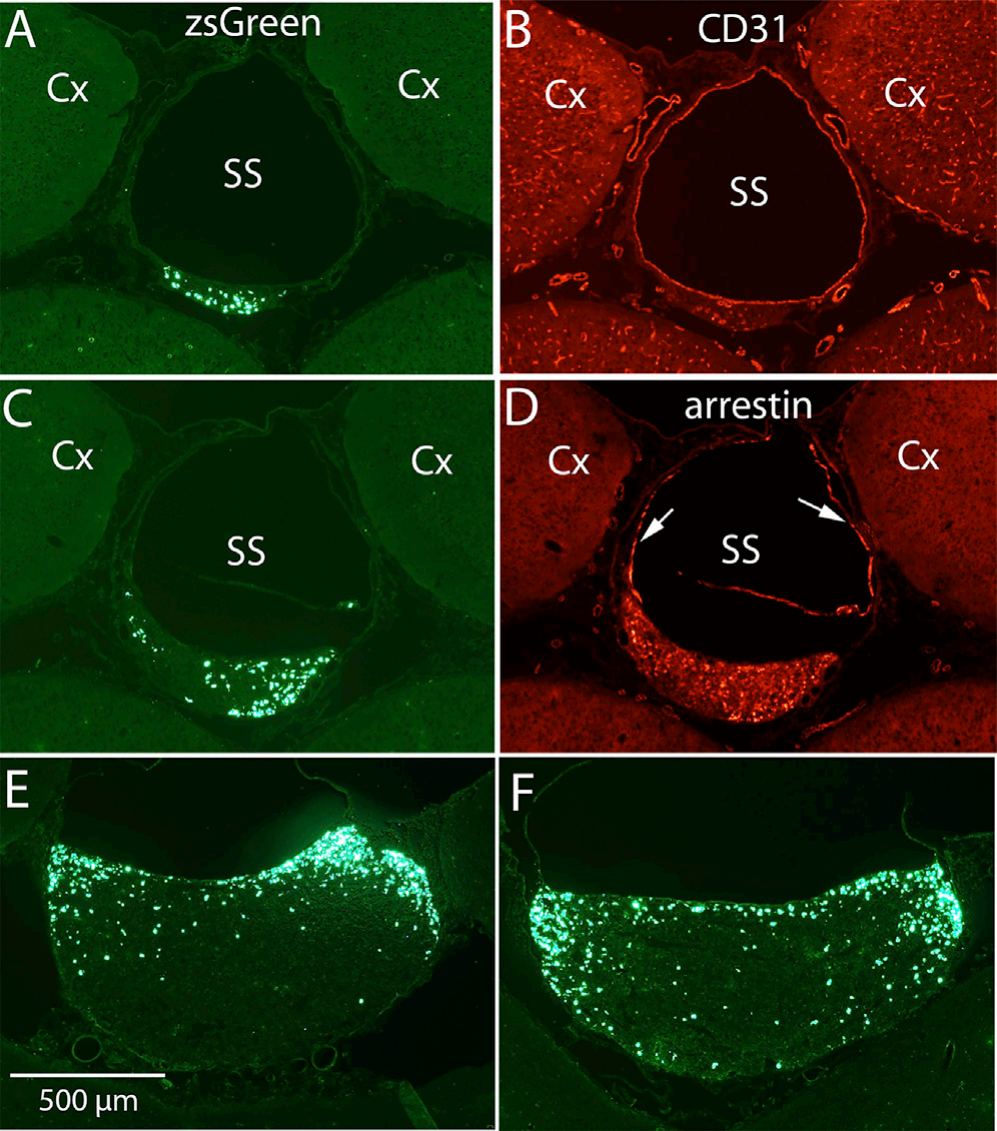
Rostrocaudal distribution of transduced cells in the pineal gland and association with the sagittal sinus (A, C, E, and F) Rostrocaudal distribution of zsGreen-positive cells in sections at approximately 500-μm intervals through the pineal gland. Note that zsGreen-positive cells are concentrated in the dorsal and lateral part of the gland near where the gland is intimately associated with the overlying sagittal sinus (SS). (B) Section in (A) immunostained for CD31, a marker for vascular endothelium. (D) Section in (C) immunostained for arrestin, a selective marker for pinealocytes. Note elongated arrestin-positive cells extending from the pineal gland proper up into the wall of the sagittal sinus (arrows). Calibration bar in (E) represents 500 μm and applies to all panels. Cx, cerebral cortex.

Examination of coronal sections taken at approximately 500-μm intervals through this pineal gland (Figures 2A, 2C, 2E, and 2F) revealed that zsGreen-positive cells were concentrated in the dorsolateral part of the pineal gland, where it is closely associated with the overlying sagittal sinus. In the most rostral part of the pineal gland near its stalk, the pineal gland is a small, crescent-shaped structure just beneath the sagittal sinus in which zsGreen-positive cells are evident (Figure 2A). Figure 2B illustrates the same section immunostained for CD31, a marker for vascular endothelium. To confirm this crescent-shaped structure as part of the pineal gland, we stained a section about 250 μm caudal to the one in Figures 2A and 2B for arrestin, a selective marker for pinealocytes, which are the resident cells of the pineal gland. Figure 2C illustrates zsGreen-positive cells in this section, and Figure 2D illustrates the same section imaged for arrestin immunohistochemistry (IHC). Specificity of labeling is demonstrated by the absence of arrestin staining in the nearby cortex. Of note, elongated arrestin-positive cells extend from the pineal gland proper up into the wall of the sagittal sinus (Figure 2D, arrows); this suggests a previously undescribed close association between pinealocytes and the vascular endothelium. The pineal gland increases in size moving caudally, and zsGreen-positive cells are concentrated dorsally and laterally in the area of the pineal gland beneath the sagittal sinus (Figures 2E and 2F).

To estimate the total number of zsGreen-positive cells in the pineal gland in Figures 1E and 1F, we counted zsGreen-positive cells in every third section of a series taken at 120-μm intervals through the ganglion and, by extrapolation, estimated the total number that would be present in sections that were not counted. Based on this extrapolation, we estimate that there are 4,750 zsGreen-positive cells in this pineal gland.

The pineal glands illustrated in Figures 1A–1C and 1F exhibited robust transduction of large numbers of cells, but in some cases, fewer zsGreen-positive cells were present. There was no obvious difference in the extent of transduction at the injection site that would explain these differences in pineal transduction. For example, there was robust transduction of the pineal glands in all three cases in Figures 1A–1C despite differences in zsGreen fluorescence at the cortical injection site.

To confirm that pineal gland labeling was not due to some peculiar property of the zsGreen reporter, we examined pineal glands from rats that received intra-cortical injections of AAV2/9-shPTEN-GFP (same injection parameters as above). In contrast with zsGreen, native GFP fluorescence was weak when intact pineal glands were visualized by epifluorescence and in un-stained sections. However, immunostaining sections for GFP revealed strong fluorescence at the injection sites in the cortex (Figure 1H), and GFP-positive cells were evident in the pineal gland in four of six cases (Figure 1I). Of note, there were fewer GFP-positive cells per section in these cases than in the cases that received AAV2/9-shPTEN-zsGreen despite the fact that the number of genome copies (GCs) delivered into the cortex was the same (five injections of 0.5 μL of the stock dilution 1.05 × 10^12^ GCs/mL).

As a technical note, detection of transduction of the pineal gland was greatly facilitated when zsGreen was the reporter rather than GFP because native zsGreen fluorescence was very bright and individual cells could be detected without immunostaining. Also, zsGreen accumulated in the nuclei of transduced cells, whereas with immunostaining, GFP fluorescence extended throughout the cytoplasm, robustly labeling neuronal dendrites and astrocyte processes. Consequently, it was difficult to distinguish and count individual labeled cells in areas of high transduction with GFP as the reporter.

#### Assessment of which cell types in the pineal gland are transduced

**Figure 3:**
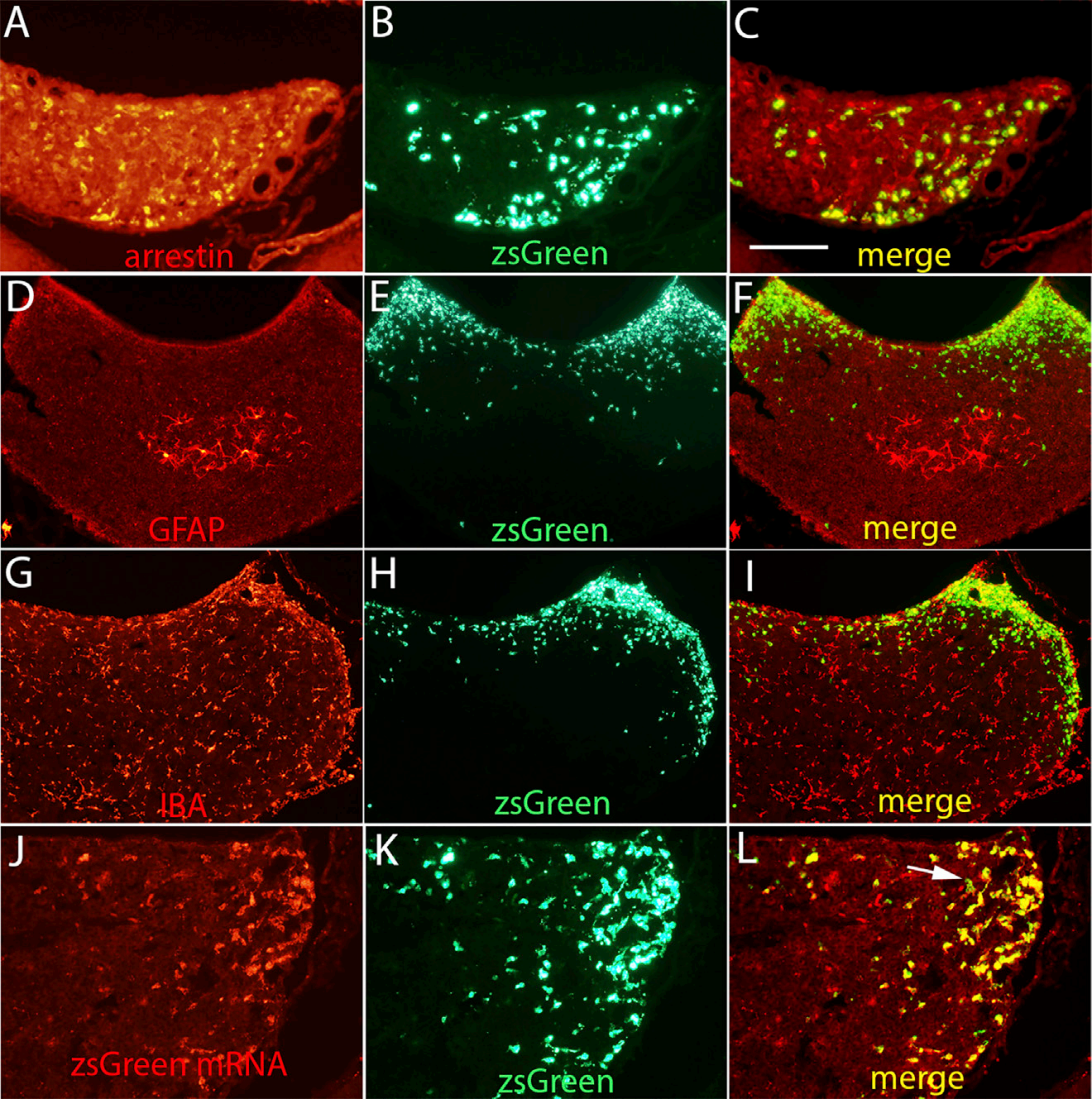
Assessment of which cell types in the pineal gland are transduced by AAV and detection of AAV mRNA by RNAscope (A and B) Higher-magnification views of arrestin-positive cells and zsGreen-positive cells from the section illustrated in Figures 2C and 2D. (C) Co-imaging for zsGreen and arrestin revealed arrestin-positive cells that contained zsGreen (yellow). (D) Immunostaining for GFAP revealed GFAP-positive cells concentrated in the core of the pineal gland. (E) zsGreen-positive cells in the same section as shown in (D). (F) Co-imaging for zsGreen and GFAP revealed no instances of co-localization of zsGreen fluorescence in GFAP-positive astrocytes. (G) Immunostaining for IBA1 revealed IBA1-positive microglia distributed throughout the pineal gland. (H) zsGreen-positive cells in the same section as in (G). (I) Co-imaging for zsGreen and IBA1 revealed no zsGreen fluorescence in IBA1-positive microglia. (J) RNAscope reveals expression of zsGreen mRNA in cells in the dorsolateral part of the pineal gland. (K) zsGreen-positive cells in the same section as in (J). (L) Co-imaging for zsGreen mRNA and zsGreen reveals extensive co-labeling (yellow). Arrow indicates two cells that are labeled for zsGreen mRNA, but not arrestin. Calibration bar in (C) represents 125 μm for (A)–(C) and (J)–(L) and 250 μm for (D)–(I).

To assess which cell types in the pineal are transduced, sections from rats that received AAV2/9-shPTEN-zsGreen were immunostained for cell-type-specific markers, including arrestin, GFAP (a marker for astrocytes), and IBA1 (a marker for microglia). Figures 3A and 3B are higher-magnification views of arrestin-positive cells and zsGreen-positive cells from the section illustrated in Figures 2C and 2D. Co-imaging for zsGreen and arrestin revealed arrestin-positive cells that contained zsGreen (Figure 3C, yellow).

Immunostaining for GFAP revealed that GFAP-positive cells were concentrated in the rostral part of the pineal gland near its stalk that connects the gland to the brain, and more caudally, in the core of the pineal gland (Figure 3D). In the most caudal region of the gland, no GFAP-positive cells were evident. In contrast, zsGreen-labeled cells were distributed in the superficial part of the pineal gland (Figure 3E). As expected from the non-overlapping distribution, co-imaging for zsGreen and GFAP revealed no instances of co-localization of zsGreen fluorescence in GFAP-positive astrocytes (Figure 3F).

Immunostaining for IBA1 revealed IBA1-positive microglia distributed throughout the pineal gland (Figure 3G). Figure 3H illustrates the distribution of zsGreen-positive cells in the same section. Co-imaging for zsGreen and IBA1 revealed no instances of zsGreen fluorescence in IBA1-positive microglia (Figure 3I). These results document that it is primarily, if not exclusively, pinealocytes that express the zsGreen reporter.

#### *In situ* hybridization to detect the vector

The presence of zsGreen-positive cells in the pineal gland could in theory reflect accumulation of zsGreen protein expressed elsewhere or the presence of the AAV vector, which is actively transcribing the fluorescent reporters. The latter is more likely, but to test this directly, we hybridized sections through the pineal gland from the same case with RNAscope probes for zsGreen mRNA expressed by the vector. Figure 3J illustrates robust labeling of cells in the pineal gland for zsGreen mRNA. The distribution of zsGreen-positive cells in the same section is illustrated in Figure 3K. Co-imaging for zsGreen mRNA and zsGreen reveals extensive co-labeling (Figure 3L). These results confirm that zsGreen fluorescence indicates the presence of the AAV vector.

### Accumulation of AAV in the pineal gland with AAV injections into the dentate gyrus

**Figure 4:**
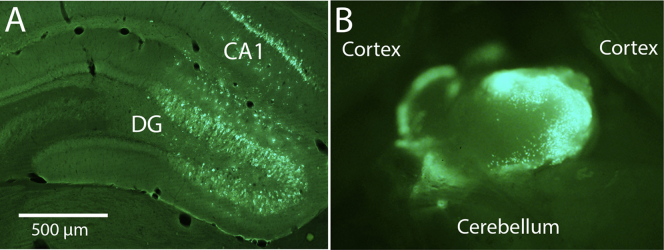
Accumulation of AAV in the pineal gland following injections into the dentate gyrus (A) zsGreen expression in the dentate gyrus with single injections of 0.3 μL of AAV2/9-shPTEN-zsGreen. The image shown was included in a previous study of synaptically driven IEG expression in PTEN-depleted dentate granule cells.^3^ Other examples of the transduction with this injection paradigm are in this article. (B) zsGreen-labeled cells in the intact pineal gland in the same case. Calibration bar represents 500 μm.

We were curious whether AAV injections into other brain regions would also lead to accumulation in the pineal gland. For this, we determined the incidence of pineal transduction in rats that received a single injection (0.3 μL) of AAV2/9-shPTEN-zsGreen or AAV2/9-shLuc-zsGreen into the hippocampal dentate gyrus. Some of the tissue came from cases included in a study of synaptically driven IEG expression in PTEN-depleted dentate granule cells.^3^ Injection sites in the dentate gyrus were marked by robust zsGreen expression (Figure 4A), with minimal labeling in other structures. The majority of rats that received AAV2/9-shPTEN-zsGreen (8/13) or AAV2/9-shLuc-zsGreen (3/5) exhibited robust zsGreen labeling of cells in the pineal gland (Figure 4B).

#### How does AAV reach the pineal gland from a distant injection site?

When injected into the brain parenchyma, AAVs are taken up by neurons and astrocytes at the injection site based on trophic specificity. Even many months post-injection, expression of AAV cargos is largely restricted to the injection site, for example, the sensorimotor cortex in the case illustrated in Figures 1A–1D and the dentate gyrus in the case illustrated in Figure 4A. Despite this localized expression, at least some AAV particles undoubtedly diffuse away from the injection site into the interstitial fluid and then the cerebrospinal fluid (CSF). Accordingly, we describe two general possibilities for how AAVs could reach the pineal gland from distant injection sites. First, AAV particles diffuse from the injection site into the CSF and then reach the pineal gland directly via the CSF or indirectly via routes and mechanisms discussed further below. This could occur at the time of the injection or progressively over time. Second, some cell type at the injection site that is transduced by the AAV subsequently migrates to the pineal gland.

#### Pineal gland transduction via the CSF?

If AAV particles diffuse from the injection site into the CSF, then one would expect some transduction of neurons and glia at a distance from the injection in areas contacted by the CSF. Examination of coronal sections from rats that received injections of AAV2/9-shPTEN-zsGreen or AAV2/9-shPTEN-GFP into the sensorimotor cortex revealed evidence consistent with this possibility.

**Figure 5:**
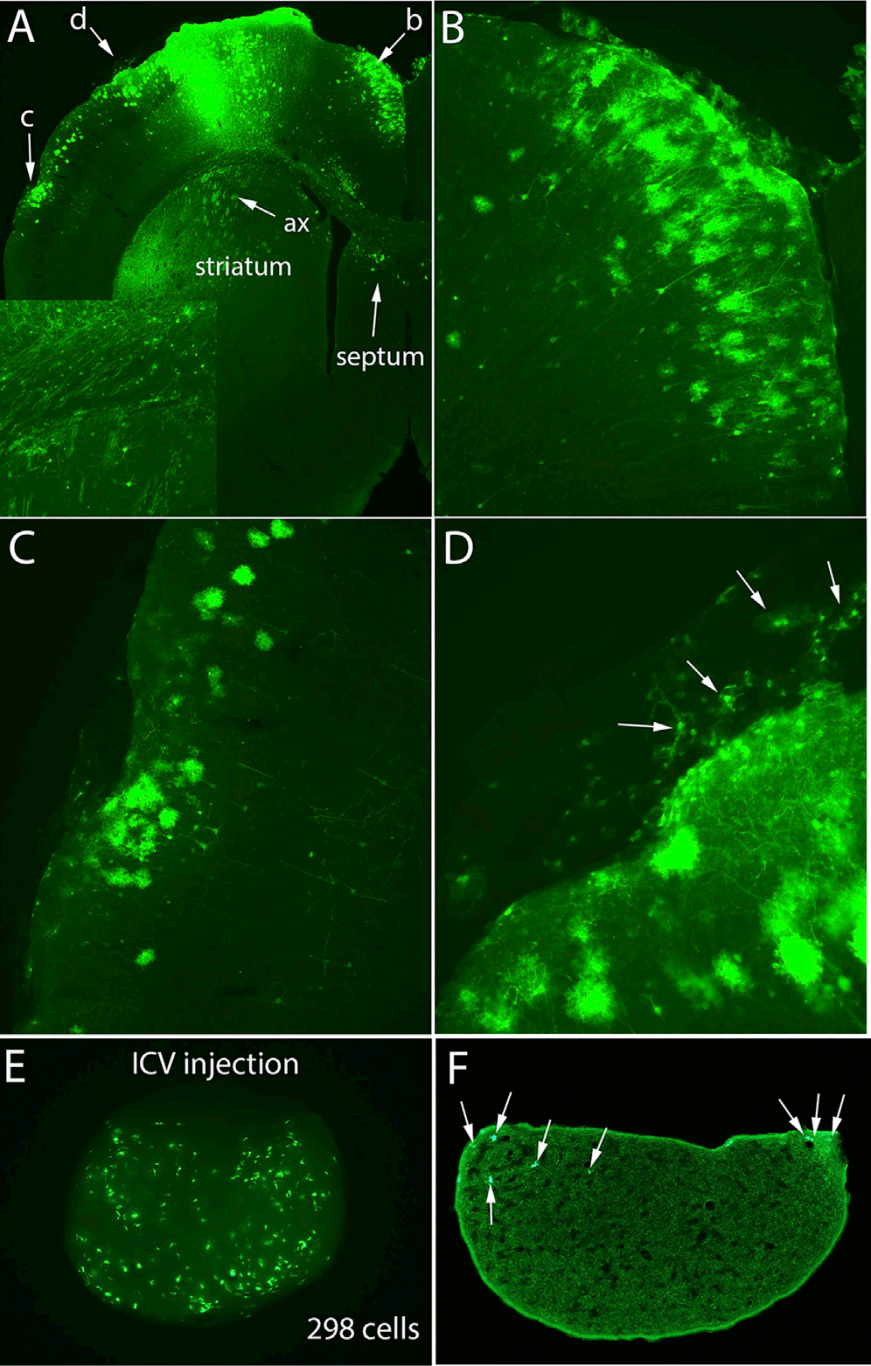
Widespread distribution of transduced cells in the cortex many millimeters from the injection site (A) Coronal section through the cortex in a rat that received AAV2/9-shPTEN-GFP reveals many GFP-positive cells at a distance from the injection site with the morphology of astrocytes along with scattered GFP-positive neurons. Inset illustrates labeled axons in the white matter and striatum. Arrows and lowercase letters indicate locations of (B)–(D). Note also a few labeled neurons and astrocytes in the dorsal part of the septal region and in the dorsomedial part of the striatum bordering the lateral ventricle (arrows). (B) GFP-labeled cells in the cortex near the midline medial to the injection site. (C) GFP-labeled cells in the cortex lateral to the injection. (D) Higher-magnification view of GFP-labeled cells in a fragment of the meninges that had detached and flattened out next to the section (arrows). (E) zsGreen-labeled cells in the intact pineal gland 9 days following injections of 0.3 μL of AAV29-shPTEN-zsGreen into the CSF at the cisterna magna. (F) zsGreen-labeled cells in a 30-μm section through the pineal gland in (E). Calibration bar in (A) represents 500 μm and applies to (A)–(C).

In sections from rats that received AAV2/9-shPTEN-zsGreen, zsGreen was concentrated at the injection site (Figure 1D), but zsGreen-positive cells were also evident in the superficial layers of the cortex lateral to the injection sites (Figure 1D). In rats that received AAV2/9-shPTEN-GFP, immunostaining for GFP revealed widespread distribution of GFP-positive cells in the cortex many millimeters from the injection site (Figure 5A). Most of the GFP-positive cells at a distance from the injection site had the morphology of astrocytes, but there were also scattered GFP-positive neurons. Figure 5B illustrates GFP-labeled cells in the cortex near the midline, and Figure 5C illustrates GFP-labeled cells in the ventrolateral cortex. In this brain, there were also a few labeled neurons and astrocytes in the dorsal part of the septum and in the dorsomedial part of the striatum bordering the lateral ventricle (Figure 5A).

The distributed sparse transduction described above would be consistent with diffusion of AAV from the injection sites via the CSF, but the question remains regarding how AAVs would reach the pineal gland. Provocative evidence comes from incidental findings from the brain illustrated in Figure 5. Our standard protocol is to remove the dura mater from brains prior to embedding, but the pia/arachnoid meninges remain partially attached. Importantly, there were GFP-positive cells within these meningeal fragments indicating transduction by AAV2/9-shPTEN-GFP. Figure 5D illustrates a fragment of meninges with GFP-positive cells that had detached and flattened out next to the section (arrows).

Together, these observations suggest the hypothesis that AAVs diffuse from the injection site in the interstitial fluid and enter the CSF and meninges. The AAVs could then travel through the meninges to the pineal gland or travel with other wastes in the CSF into the dural sinuses and then cross over to the closely associated pineal gland because of the absence of a blood-brain barrier. Whatever the route, our data suggest efficient tropism of AAV for pinealocytes.

#### Transduction of cells in the pineal gland with direct injections of AAV into the CSF

With local injections into the brain parenchyma, uptake by cells at the injection means that only some of the AAV diffuses into the CSF. Thus, if AAVs injected into the parenchyma reach the pineal gland via the CSF, then direct injections into the CSF could lead to even more pineal transduction. To assess this, we injected AAV2/9-shPTEN-zsGreen into the CSF at the cisterna magna at the same volume and concentration as the injections into the dentate gyrus (0.3 μL with 3 × 1E7 GCs). Rats were allowed to survive for 9 days or 8 weeks. By 9 days after ICV injection, zsGreen-labeled cells were evident in intact un-sectioned pineal glands (Figure 5C). To obtain this image, we removed the pineal gland from the brain and connective tissue, embedded it in agarose, and imaged with epifluorescence. Because there were fewer zsGreen-positive cells than in the cases illustrated in Figures 1 and 4B, it was possible to actually count transduced cells in this intact gland; 298 zsGreen-positive cells were present.

In cross-sections through this pineal gland (section thickness, 20 μm), four to eight zsGreen-positive cells were seen per section; Figure 5D illustrates one section through the main part of the gland with eight zsGreen-positive cells. This is approximately the number that would be expected based on the counts from the intact pineal gland. Sixty sections at 20 μm were required to through-section this pineal gland. Dividing the total number in the ganglion (298) by 60 sections yields a prediction of an average of 5 zsGreen-positive cells per section. It should be noted that this is an approximate calculation, which fails to account for counting errors because of cells that are bisected by the section. Nevertheless, the rough correspondence indicates that counts per section can provide a reasonable estimate of the total number present in the intact ganglion and vice versa.

Of note, approximately the same number of zsGreen-positive cells was seen in sections through the pineal gland of a rat that survived for 2 months post-ICV injection (this pineal gland was not imaged by epifluorescence prior to sectioning). These results document that AAVs can reach the pineal gland when injected at the cisterna magna but suggest that direct injection into the CSF may actually be less efficient than with intra-parenchymal injections.

#### Possible migration of transduced cells from the injection site to the pineal gland?

**Figure 6:**
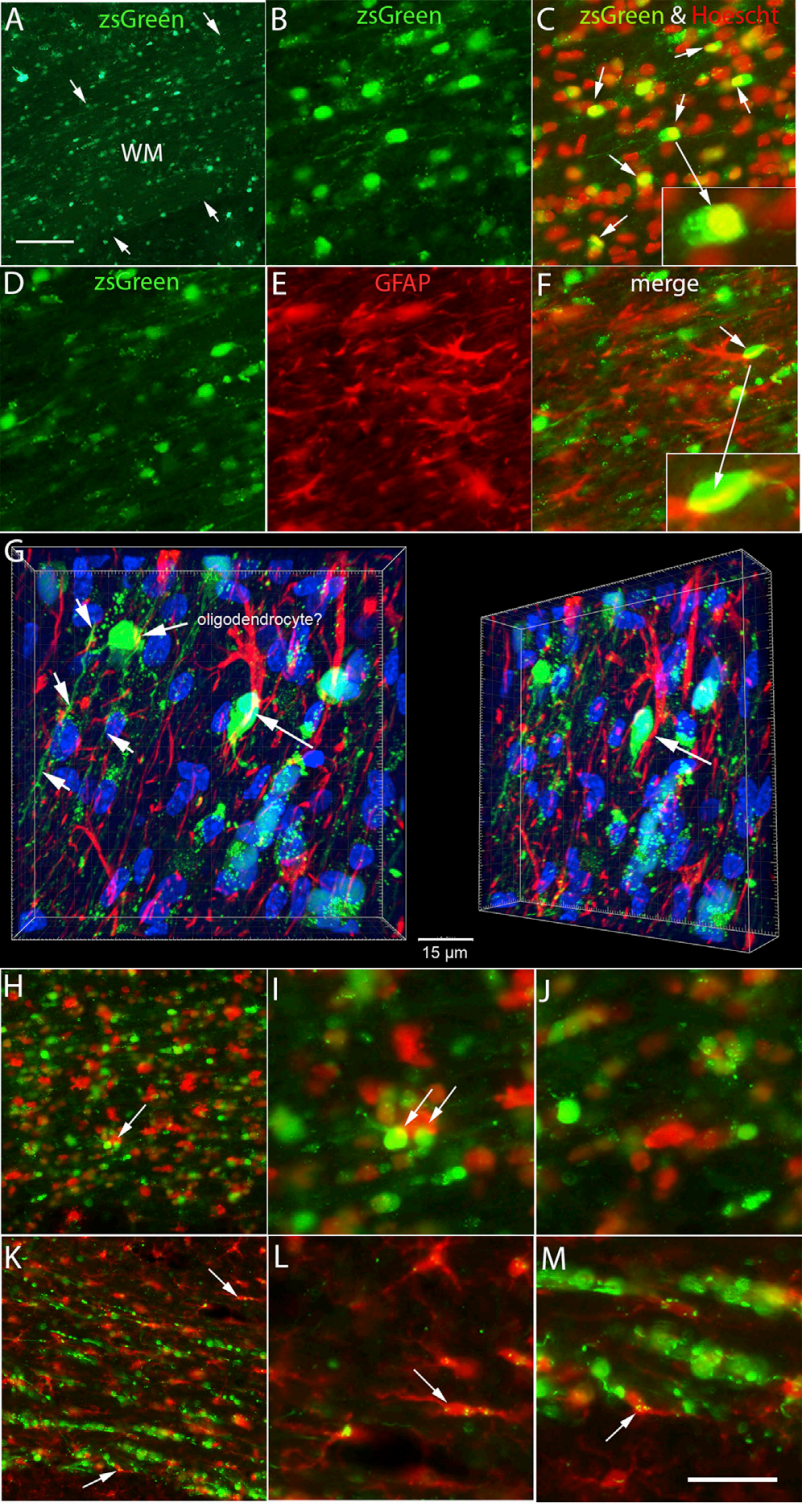
Presence of zsGreen in different cell types in the subcortical white matter (A) Example of zsGreen in subcortical white matter in a case in which white matter labeling was especially prominent. Arrows indicate the boundary between the subcortical white matter and the overlying cortex and underlying striatum. (B) Higher-magnification view of zsGreen-positive cells in the white matter. (C) Merged image of Hoechst staining converted to red and zsGreen. Note that most large zsGreen-positive granules correspond to nuclei co-stained for Hoechst, and small zsGreen granules are in the cytoplasm surrounding Hoechst-positive nuclei. (D) zsGreen granules in section stained for GFAP. (E) Immunostaining for GFAP in the same location as (D). (F) Merged image of zsGreen and GFAP. Inset illustrates a GFAP-positive process that overlaps with a GFP-positive granule without actual co-localization. (G) Confocal image of the area illustrated in (F) with zsGreen, GFAP-positive cell in red and nuclei in blue (Hoechst); the GFP-positive cell in the inset in (F) overlies a GFAP-labeled process from a nearby astrocyte (see rotated image in right panel). A zsGreen-positive cell with the morphology of a white matter myelin-forming oligodendrocyte is indicated. (H and I) Imaging for zsGreen and immunostaining for APC/CC1 (red). Arrows indicate zsGreen-labeled cells overlapping with an APC/CC1-labeled oligodendrocyte. (K–M) Imaging for zsGreen and immunostaining for IBA1 (red). Arrows indicate small zsGreen granules within IBA1-positive microglial cells. Calibration bar in (M) represents 100 m for (B)–(F), (I)- (M) .

The other possible mechanism for transduction of the pineal gland is that some cell type at the injection site that is transduced by the AAV subsequently migrates to the pineal gland. In addition to zsGreen- and GFP-labeled axons (depending on vector) in the subcortical white matter (Figure 1D), there were also zsGreen- and GFP-labeled granules in the white matter, especially in cases in which the injection track extended into the white matter. Figure 6A illustrates one case in which zsGreen granules in the white matter were especially prominent.

To explore whether zsGreen granules in white matter might be in a cell type that could migrate away from the injection site and eventually reach the pineal gland, we immunostained sections with Hoechst to stain nuclei and with antibodies that are cell-type-specific markers. As illustrated in Figure 6C (merged image of Hoechst in red and zsGreen), most of the large zsGreen-positive granules were in nuclei that co-stained for Hoechst, with smaller granules present in the cytoplasm surrounding Hoechst-positive nuclei.

Astrocytes are one cell type that could proliferate and potentially migrate, and so we stained sections for GFAP and imaged sections for GFAP and zsGreen. Although there were some instances of overlap of zsGreen (Figure 6D) and GFAP immunoreactivity (red, Figure 6E), these were situations in which a GFAP-positive process overlapped with a zsGreen-positive granule without actual co-labeling (see enlarged image in inset in Figure 6F). Confocal microscopy confirmed this conclusion (Figure 6G); the zsGreen P-positive cell in the inset in Figure 6F overlies a GFAP-labeled process from a nearby astrocyte that can clearly be distinguished when the image is rotated (Figure 6G, image on right). Thus, neither the large-nuclei-size zsGreen granules nor the small granules in white matter were localized within GFAP-positive astrocyte cell bodies or processes.

The confocal image also reveals another zsGreen-positive cell with the morphology of a white matter myelin-forming oligodendrocyte (Figure 6G). zsGreen-positive processes can be seen extending from the cell body and then along the plane of the axons in the white matter (small un-labeled arrows). Although this cell has the morphology of an oligodendrocyte, immunostaining other sections for APC/CC1 and co-imaging for APC/CC1 and zsGreen did not reveal examples of co-labeling (Figures 6H–6J). There were examples of overlap of zsGreen and IBA1-positive cells (Figure 6I, arrows), but there were no instances of actual co-localization. Because the main focus of our search was for cells that might migrate and carry AAVs to the pineal gland, and oligodendrocytes do not migrate in the mature CNS, we did not pursue the question of possible zsGreen-labeled oligodendrocytes further.

Microglia are another cell type that theoretically could take up AAV and then migrate, although previous studies indicate limited AAV transduction of microglia.^4^ Nevertheless, to assess this possibility, selected sections were immunostained for IBA1, a marker for microglia, and co-imaged for zsGreen and IBA1 (Figures 6K–6M). None of the zsGreen-positive nuclei were within IBA1-positive microglia, but there were a few IBA1-positive cells that contained small zsGreen-positive granules (Figures 6L and 6M, arrows).

Although our assessment with cell-type-specific markers did not reveal the identity of the cells with zsGreen-positive nuclei in subcortical white matter, there was transduction of astrocytes in the gray matter of the cortex at a distance from the injection and microglia in the subcortical white matter. In theory, either astrocytes or microglia could migrate away from the injection site and eventually reach the pineal gland with their AAV cargo. Given the small number of transduced microglia, it seems unlikely that these could deliver sufficient AAVs to the pineal gland to account for the labeling that is seen. Also, the absence of transduced astrocytes or microglia in the pineal gland means that any transduced carrier that reaches the pineal must either migrate away or undergo cell death after delivering the AAV cargo to the pineal gland.

### Transduction of cells in the pineal gland following AAV2-retro/Cre injections into the spinal cord

We were curious whether there would be similar accumulation in the pineal gland with AAV injections into more distant sites in the nervous system, specifically the spinal cord. To assess this, we examined brains from tdT-reporter mice that received injections of AAV2-retro/Cre into different levels of the spinal cord (vertebral level T9 and lumbar vertebral level 2 [L2]) to retrogradely transduce the cells of origin of spinal pathways in the brain. Some of these mice were included in a study defining the topographic organization of corticospinal tract projections;^2^ other mice were from pilot studies using AAV2-retro/Cre to transduce cortical motoneurons after spinal cord injury.

**Figure 7:**
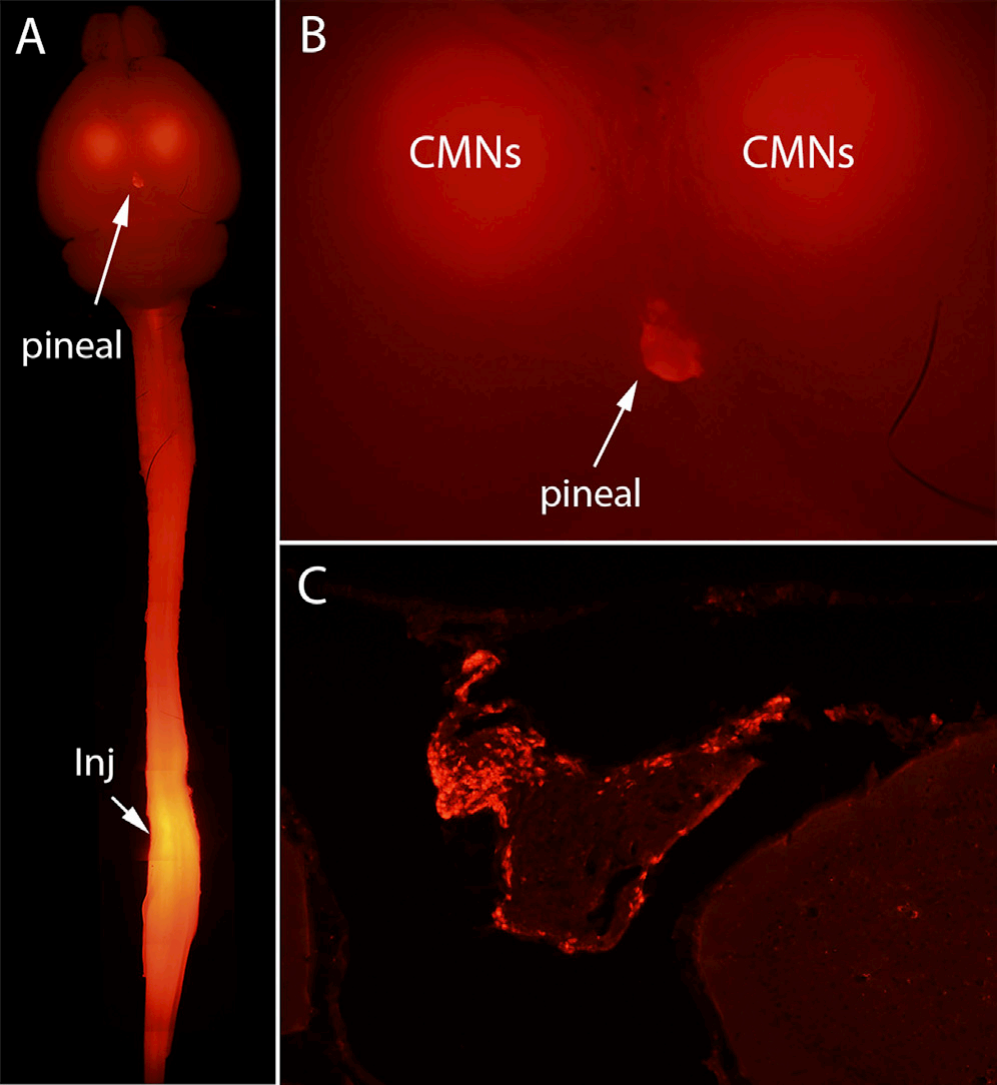
TdT labeling in the pineal gland with AAV/Cre injections into the spinal cord of tdT reporter mice (A) Intact brain and spinal cord with fluorescence epi-illumination. The injection site in the spinal cord and a cloud of retrogradely transduced tdT-positive cortical motoneurons (CMNs) are visible, along with tdT labeling in the pineal gland. (B) Higher-magnification view of tdT labeling in the pineal gland. (C) TdT-labeled cells in a cross-section through the pineal gland of a different mouse that received AAV/Cre injections rostral to a crush injury at T9 and survived 2 months post-injury. tdT-positive cells with the morphology of pinealocytes are evident in the periphery of the pineal gland.

With AAV2-retro/Cre injections into the spinal cord, tdT fluorescence indicating Cre-mediated recombination was evident in the pineal glands of many mice. An example of tdT labeling with AAV2-retro/Cre injections at T9 is illustrated in Figures 7A and 7B. Figure 7A illustrates the intact brain and spinal cord of one case using fluorescence epi-illumination. The injection site in the spinal cord and a cloud of retrogradely transduced tdT-positive cortical motoneurons that give rise to corticospinal projections to T9 are evident, along with tdT labeling in the pineal gland. Figure 7B illustrates a higher-magnification view of tdT labeling in the brain. Figure 7C illustrates a section through the pineal gland of a different mouse that received AAV2-retro/Cre injections rostral to a crush injury at T9 and survived 2 months post-injury. As above, tdT-positive cells with the morphology of pinealocytes are evident in the periphery of the pineal gland.

#### Intracerebroventricular (i.c.v.) injections of different AAV serotypes

Our results document robust transduction of cells in the pineal gland with intracranial and i.c.v. injections of AAV2/9 and intra-spinal cord injection of AAV2-retro, which was developed through directed evolution to be efficiently transported in a retrograde fashion along axons. As an initial step toward assessing transduction by different AAV serotypes, we injected different AAVs into the CSF at the cisterna magna, including AAV2/9-shPTEN-zsGreen (described above in Figure 5E), AAV2/9-GFP (different from the AAV2/9-shPTEN-GFP described above in Figures 1H and 1I), and commercially available AAV2-GFP and AAV9-GFP. Immunostaining sections through the pineal gland for GFP revealed a few transduced cells with each vector. Of note, however, the number of cells transduced by what we considered to be the positive control (AAV2/9-GFP) was less than what is observed in other experiments described above. We did not pursue this question further because it will likely be necessary to optimize conditions (GC dose, site of injection, survival time, and perhaps method of detecting transduced cells) to provide a comprehensive picture of differences between AAV subtypes in their ability to transduce the pineal gland.

## Discussion

Here, we report an unexpected efficient accumulation of AAV vectors in the pineal gland after injections into different sites in the brain and spinal cord or the CSF. We confirm that it is actually the AAV2/9-PTEN-zsGreen vector that is present using RNAscope to detect the mRNA for zsGreen. In the case of tdT fluorescence in the pineal gland in reporter mice that received AAV-retro/Cre injections into the spinal cord, tdT expression almost certainly indicates transduction of pinealocytes by AAV-retro/Cre.

How AAVs reach the pineal gland from the different injection sites is not firmly established, but our results are most consistent with the idea that some AAV particles diffuse from the injection site via the interstitial fluid, enter the CSF, and are then disposed of via the same routes as other waste material. The textbook view is that the CSF carries waste products away from the central nervous system and drains into the sagittal sinus. In humans, CSF drainage into the sinus is thought to occur at arachnoid granulations. However, recent studies have documented an alternate route for disposal of waste products via the lymphatic system of the meninges.^5^ Thus, AAVs could either travel through the meninges to the pineal gland or could enter the dural sinuses and then cross over to the intimately associated pineal gland because of the absence of a blood-brain barrier. This possibility is supported by the fact that direct injections of AAV2/9-shPTEN-zsGreen into the CSF via the cisterna magna did lead to transduction of cells in the pineal gland. It cannot be excluded, however, that with focal injections into the brain parenchyma, some type of cell takes up AAV at the injection site and then migrates to the pineal gland. In this case, the AAV would still have to be released by a putative delivery cell and taken up by pinealocytes in the pineal gland, or the delivery cell would have to trans-differentiate into a pinealocyte to account for the fact that virtually all of the transduced cells in the pineal gland are pinealocytes.

Arrival of AAV via axonal projections from input pathways is a theoretical possibility but seems very unlikely. The pineal gland receives projections from the suprachiasmatic nucleus, which is involved in circadian control of melatonin production, but we have not seen evidence of AAV transduction of neurons in the suprachiasmatic nucleus with injections into the cortex or dentate gyrus. There are also projections to the pineal gland from the superior cervical ganglion, but this route seems even less likely because this would require that AAVs somehow reach the superior cervical ganglion from the injection sites in the brain and spinal cord. Also, transduction via axons would then require some form of *trans*-synaptic transport of the AAV from axons to pinealocytes. Although transneuronal transduction has been reported for other AAVs such as AAV1,^6^ AAV2 is not thought to be transported efficiently from pre- to postsynaptic neurons, and we have not seen any evidence of this in our studies. Thus, taken together, the combined evidence suggests that the most likely mechanism involves leakage of AAVs from the injection sites into the CSF and then efficient uptake of AAV by pinealocytes.

Transduction of the pineal gland with injections into the spinal cord is of particular interest because of the distance involved. Delivery through the CSF is possible because CSF flows back between the spinal cord to the brain, and the canonical view is that CSF and wastes it contains are removed into the venous drainage of the brain. Alternatively, intra-spinal cord injections of AAV-retro/Cre lead to retrograde transduction of thousands of neurons in the cortex that project to the spinal cord,^2^ and it is possible that AAV-retro/Cre is released from transduced brain neurons into the interstitial fluid and then the CSF. Further studies will be required to explore these possibilities.

The proposed mechanism above is consistent with known aspects of pineal gland functions but also suggests some additional provocative ideas. The pinealocytes in the pineal gland produce and release melatonin, and circadian rhythmicity of release is controlled by neural inputs from the supra-chiasmatic nucleus of the hypothalamus. Melatonin is released into the bloodstream, and the intimate association between the pineal gland and the overlying sagittal sinus suggests the path to the venous outflow. This intimate association between the pineal gland and the venous sinus also positions the gland in an ideal location to sample the effluent from the brain. It would make perfect biological sense if the pineal gland, which is important for controlling sleep, was poised to increase production of the sleep-initiating hormone melatonin in response to toxic waste material in the brain effluent in the sagittal sinus. It would not be surprising if such a toxin-sampling system was especially attuned to detect viral capsids and viral proteins in the main venous drainage from the brain.

It is unknown whether accumulation of AAV vectors in the pineal gland is a general property of AAVs or is unique to certain serotypes. Here, we document transduction with AAV2/9 and AAV2-retro, which was developed through directed evolution to be efficiently transported in a retrograde fashion along axons. In a limited study of transduction by different AAV serotypes following i.c.v. injections, we also observed some transduction with commercially available AAV2/GFP and AAV9/GFP vectors. The key question, however, is not whether standard AAV serotypes accumulate in the pineal gland but rather whether the serotypes developed through rational design or directed evolution that are being used as therapeutic candidates do. Future studies of engineered AAVs will provide insights into properties of capsid proteins that determine the ability of AAVs to efficiently transduce cells in the pineal gland.

### Functional implications and considerations for AAV-based therapies

Accumulation of AAV in the pineal gland after injections into different sites in the CNS has potential implications for AAV-based therapies. One question is whether “off-target” accumulation of therapeutic AAVs and expression of their gene-modifying cargos in pinealocytes are a safety concern. The canonical function of the pineal gland is to produce and release melatonin, which is regulated by the light/dark cycle via input from the supra-optic nucleus of the hypothalamus, as well as via inputs from the sympathetic nervous system via the superior cervical ganglion. In turn, the circadian cycle of melatonin release plays an important role in regulating the sleep/wake cycle. Accordingly, any gene-modifying cargos expressed by AAVs could disrupt the physiological controls on melatonin release and thus disrupt the sleep/wake cycle. For example, recent studies suggest that pineal gland dysfunction contributes to sleep disturbances in Alzheimer’s disease.^7^

Of potentially greater concern is the possibility of a pineal neoplasm or tumor, such as pineocytoma, pineoblastoma, or primary pineal malignant melanoma.^8^ Pineal tumors are challenging for neurosurgery because of their location, and even a low-probability risk for a pineal tumor is a safety concern, although new surgical approaches are being developed that improve outcomes.^9^ On the other side, efficient accumulation of AAVs in the pineal gland could provide a way to deliver therapeutic cargos to the pineal gland to treat pineal tumors. Our findings suggest the need for further studies of the mechanisms and significance of the unexpected accumulation of AAV in the pineal gland. In addition, it may be prudent going forward to include the pineal gland in routine screens for off-target accumulation of therapeutic candidate AAVs that are delivered into the CNS, as well as AAVs that are delivered systemically, because the pineal gland is one of the brain structures that lacks a blood-brain barrier.^10^

## Materials and methods

### AAV vectors

Initial studies in rats were done using AAV2/9 vectors designed to delete PTEN via expression of short hairpin RNA (shRNA).^11^ These express shRNA against PTEN from the human U6 promoter and either a zsGreen reporter protein from the CMV promoter (AAV2/9-shPTEN-zsGreen) or GFP (AAV2/9-shPTEN-GFP). We also used a control vector expressing shRNA against luciferase (AAV2/9-shLuc-zsGreen). These vectors were produced by the University of Pennsylvania Vector Core. Studies involving tdT reporter mice used AAV2-retro/Cre obtained from Addgene (catalog number [cat#] 24593). AAV2-retro is a “designer” variant modified through directed evolution that differs from standard AAV2 in that it is efficiently transported in a retrograde direction from axon terminals to neuronal cell bodies.^12^ We also assessed pineal transduction following i.c.v. or intracranial injection of AAV2/GFP, AAV8/GFP, and AAV9/GFP vectors from Addgene (cat# 37825).

#### Animals and surgery

Experimental animals were adult female Fisher 344 or female Sprague-Dawley rats and transgenic tdT reporter mice, including Rosa^tdTomato^ mice with a lox-P flanked STOP cassette that prevents expression of tdTomato (obtained originally from Jackson Labs and maintained in our breeding colony for several generations), and *Pten*^*loxP/loxP*^/Rosa^tdTomato^ transgenic mice that we generated by crossing *Pten*^*loxP/loxP*^ with Rosa^tdTomato^ mice. Rosa^tdTomato^ mice have a C57BL/6 genetic background; *Pten*^*loxP/loxP*^/Rosa^tdTomato^ transgenic mice are of mixed genetic background (C57BL/6 and 129Sv). Both male and female mice were used for studies with AAV-retro and were at least 90 days of age at the time of AAV2-retro/Cre injections. Some studies used Fisher 344 transgenic rats from our breeding colony that express GFP. All procedures were approved by the Institutional Animal Care and Use Committee (IACUC) at the University of California Irvine.

Some of the tissue used in this study is from rats used in previous studies,^3^ with additional animals prepared using the same procedures. Details on experimental procedures are summarized below.

#### Unilateral injections of AAV2/9 vectors into the sensorimotor cortex

For intracortical injection, vectors were diluted to a concentration of 1.05 × 10^12^ GCs/mL. Rats were anesthetized with isoflurane (2%–3%) and placed in a stereotaxic device. The scalp was incised, and burr holes were drilled in the skull. Using a Hamilton syringe tipped with a pulled glass pipette, we made a total of 5× 1-μL injections of AAV2/9-shPTEN-zsGreen, AAV2/9-shLuc-zsGreen, or AAV2/9-shPTEN-zsGFP (coordinates with respect to bregma: 2.5 lateral/2.0 posterior; 2.5 lateral/1.0 posterior; 3.2 lateral/1.0 posterior; 2.5 lateral/0 posterior; and 3.2 lateral/0 posterior, all at a depth of 0.8 mm) for a total of 5 × 10^9^ genome copies.

The initial discovery of pineal transduction was from rats that received complete crush injuries of the spinal cord at T9 and injections of AAV2/9-shPTEN-zsGreen or AAV2/9-shLuc-zsGreen into the sensorimotor cortex. Some rats also received transplants of GFP-labeled NSCs into the injury cavity as described previously.^13^ After completing the surgical procedures, rats were placed on a water-jacketed heating pad until fully recovered from the anesthetic. Hindlimb motor function was assessed over a 4-month survival interval and 4 months post-surgery, rats were euthanized with Fatalplus and perfused transcardially with 4% paraformaldehyde (PFA) in 0.1 M phosphate buffer (4% PFA).

Follow-up analyses used tissue from rats in three studies done for other purposes, as well as rats prepared specifically for the analysis of AAV in the pineal gland. One study used tissue from a study assessing the consequences of PTEN knockdown on IEG expression in cortical neurons (for details, see Steward et al.^3^). Ten adult male Fischer 344 rats received AAV2/9-shPTEN-zsGreen and 10 received the control vector (AAV2/9-shLuc-zsGreen). Rats were allowed to survive for 2 months post-AAV injection. This study also involved three adult male Fischer 344 rats that received intracortical injections of AAV2/9-shPTEN-GFP (same injection parameters as above). We also analyzed tissue from rats that received cortical injections of AAV2/9-shPTEN-zsGreen (n = 26) or AAV2/9-shLuc-zsGreen (n = 24) at the time of a dorsal hemisection injury of the spinal cord at C5. This set of rats survived for 4 months post-injection.

#### AAV2/9-shRNA injections into the dentate gyrus

We also analyzed tissue from rats in a study of consequences of PTEN deletion in the hippocampal dentate gyrus.^3^ Male and female Fisher 344 rats were prepared for AAV injection surgery as described above, except that a single burr hole was made in the skull over the dorsal hippocampus (3.5 posterior to bregma, 1.8 lateral). A single injection of 0.3 μL of AAV2/9-shRNA (total of approximately 3 × 10^8^ genome copies) was made at a depth of 2.8 mm to target the dentate gyrus. Fifteen female rats received AAV2/9-shPTEN-zsGreen; six rats received the control vector (AAV2/9-shLuc-zsGreen). Rats were allowed to survive for 2–5 months post-AAV injection and were then used for acute neurophysiological studies.^3^ After completion of neurophysiological procedures, rats were euthanized and perfused transcardially with 4% PFA.

#### i.c.v. injections

Adult female Sprague-Dawley rats (at least 8 weeks of age) received injections of different AAV vectors into the CSF via the cisterna magna. Rats were prepared for surgery as described above, and injections were performed using a method previously described for canula implantation ^14^. The head and neck were shaved, and the surgical site was swabbed with povidone iodine and ethanol. The rat was positioned with the head at a 135-degree angle with the body, a sagittal incision was made in the occipital crest, and muscles were bluntly dissected to expose the cisterna magna. Vectors were injected at a concentration of 1E10 GCs/mL using a 10-μL Hamilton syringe with a pulled-glass pipette tip. The tip of the glass pipette was inserted into the atlanto-occipital membrane, and 0.3 μL (3 × 1E7 GCs) was injected. Rats were euthanized and perfused with 4% PFA 10 days or 6 weeks post-ICV injection.

#### Intra-spinal cord injections of AAV-retro/Cre in Rosa^tdTomato^ and Pten^loxP/loxP^/Rosa^tdTomato^ mice

We also analyzed tissue from adult male and female transgenic mice that received bilateral injections of AAV2-retro/Cre (0.3 μL each) at T9 or L2. Some of these mice were included in a study of the topography of cortico-spinal projections to the spinal cord.^2^ Mice were anesthetized with isoflurane, and the spinal cord was exposed by laminectomy. Bilateral injections were made using a Hamilton microsyringe at 0.5 mm lateral to the midline at a depth of 0.5 mm. Three to four weeks post-injection, mice were transcardially perfused with 4% PFA in 0.1 M phosphate buffer (4% PFA), and brains and spinal cords were dissected, post-fixed in 4% PFA overnight, and stored in buffer at 4°C.

### Tissue preparation

Prior to histological preparation, intact brains and spinal cords from rats that received AAV2/9-shPTEN-zsGreen or AAV2/9-shLuc-zsGreen were examined by fluorescence epi-illumination using an Olympus AX80 microscope; images were taken at 2× and tiled to create a complete image of the brain. For tdT reporter mice that received AAV2-retro/Cre injections into the spinal cord, brains and spinal cords were removed intact. Brains were placed on a glass slide and illuminated by epifluorescence; images were taken at 2× and tiled to create a complete image of the brain and spinal cord.

#### Histology and immunocytochemistry

Brains were cryoprotected in 27% sucrose and frozen in OCT using a mixture of dry ice and 100% ethanol. Brains were sectioned in the coronal plane by cryostat collecting sets of sections at 240- or 480-μm intervals, except for the region containing the pineal gland for which every section was collected. For rats that received injections of AAVs expressing zsGreen, one set of sections was mounted without staining to visualize zsGreen fluorescence. Other sections were immunostained as described below. For rats that received injections of AAVs expressing GFP, sets of sections were immunostained for GFP and other cell-type-specific markers as described below.

#### Immunostaining for cell-type-specific markers

Selected sections were immunostained for cell-type-specific markers, including GFAP for astrocytes, APC/CC1 for oligodendrocytes, IBA1 for microglia, and arrestin, also known as antigen S, a selective marker for pinealocytes and platelet-endothelial cell adhesion molecule-1 (PECAM-1), also known as CD31, which recognizes a transmembrane glycoprotein adhesion molecule expressed by vascular endothelial cells. Sections from rats that received AAV2/9-shPTEN-GFP were also immunostained for GFP. For immunostaining, floating sections were blocked for 1–2 h in TBS with 5% normal donkey serum and 0.3% Triton X-100 and then incubated overnight in primary antibodies diluted in blocking buffer. For immunostaining sections that were mounted on microscope slides, the antigen retrieval step involved placing the slides with sections in a Coplin jar with buffer at 95°C for 5 min.

Primary antibodies were rabbit anti-GFP (cat# A-11122, RRID: AB_221569; Life Technologies); mouse anti-GFAP 1:1,000 (cat# G-3893, RRID: AB_477010; Sigma-Aldrich); mouse anti-APC/CC1 1:250 (cat# OP80-100UG, RRID: AB_2057371; Oncogene); rabbit anti-IBA1 1:1,000 (Cat# 019-19741, RRID: AB_839504; FUJIFILM Wako Shibayagi); rabbit anti-S-arrestin 1:1,000 (Cat# PA1-731, RRID: AB_2183220; Thermo Fisher); and goat anti-CD31/PECAM1 1:40 (Cat# AF3628, RRID: AB_2161028, generous gift from Dr. Yama Akbari; R&D Systems), which recognizes a transmembrane glycoprotein adhesion molecule expressed by vascular endothelial cells and is a useful marker for CNS vasculature.

Floating sections were rinsed and incubated in 250× dilutions of secondary antibodies in blocking solution as follows: for anti-GFP and anti-S-arrestin biotin-SP donkey anti-rabbit (cat# 711-066-152, RRID: AB_2340594; Jackson ImmunoResearch Laboratories); for anti-APC/CC1 biotin-SP donkey anti-mouse (Cat# 715-066-151, RRID: AB_2340788; Jackson ImmunoResearch Laboratories); for anti-CD-31/PECAM1 rabbit anti-goat biotinylated IgG (Cat# BA-5000, RRID: AB_2336126; Vector Laboratories); for anti-GFAP donkey anti-mouse Alexa Fluor 555 (Cat# A-31570, RRID: AB_2536180; Thermo Fisher Scientific); and for anti-IBA1, donkey anti-rabbit Alexa Fluor 555 (Cat# A-31572, RRID: AB_162543; Thermo Fisher Scientific). For GFP, S-arrestin, APC/CC1, and CD31/PECAM1, sections were incubated in ABC (cat# PK-6100; Vector Laboratories) and then reacted for catalyzed reporter deposition (CARD) amplification to generate tyramide-Cy3 or fluorescein isothiocyanate (FITC; only for GFP). Sections were then mounted on gelatin-subbed slides, some were stained for Hoechst 33258, and then sections were coverslipped using Vectashield.

#### *In situ* hybridization

To detect the AAV2/9-shPTEN-zsGreen vector in tissue, we used RNAscope Multiplex Fluorescent V2 assay kit from Advanced Cell Diagnostics (ACD; cat# 323100; Newark, CA, USA) to detect the presence of the mRNA for zsGreen (cat# 461251; ACD). Brains from rats perfused with PFA as above were cryo-protected in 27% sucrose, embedded in OCT, and snap frozen in a bath of crushed dry ice and ethanol. Cryostat sections were thaw mounted onto Superfrost Plus slides and allowed to air-dry overnight.

Hybridization steps followed the protocol of the RNAscope Multiplex Fluorescent V2 assay kit. Slides were washed in 1× PBS, fixed in 4% PFA in 1× PBS at 4°C for 15 min, washed 2× with PBS, and dehydrated through graded ethanols. Slides were air-dried, baked at 60°C for 30 min, then re-hydrated in 1× PBS. Sections were treated with hydrogen peroxide solution from the kit for 10 min at room temperature, washed with MilliQ water, then treated in the target retrieval solution included in the kit at 100°C for 5 min. The slides were washed in MilliQ water, placed in 100% ETOH for 3 min, and dried at 60°C for 5 min. A 0.75-inch × 0.75-inch box was drawn around the sections using an ImmEdge hydrophobic barrier pen. Protease III solution from the kit was applied to the sections, and the slides were incubated at 40°C for 30 min. Slides were washed in MilliQ water twice, then hybridized with the ready-to-use zsGreen probe at 40°C for 2 h. Slides were washed 2× with wash buffer included with the kit, then processed through a series of amplifications provided with the kit: Amp1, Amp2, and Amp3 for 30, 30, and 15 min, respectively, at 40°C with washes following each Amp. Slides were then incubated in HRP-C1 at 40°C for 15 min, washed, and then reacted in a 1:1,500 dilution of TSA Plus FITC in ACD dilution buffer for 30 min at 40°C. Slides were stained briefly in a DAPI solution from the kit and coverslipped with ProLong Diamond (#P36970; Life Technologies, Carlsbad, CA, USA).

#### Image manipulation

Color images were taken with an Olympus AX80 upright microscope with illumination and filters appropriate to the fluorophore to assess double labeling. Images were imported into ImageJ with auto contrast adjustment and merged. Images of Hoechst labeling were imported into ImageJ and converted to red to assess double labeling for GFP and Hoechst.
